# Research on the function of epigenetic regulation in the inflammation of non-alcoholic fatty liver disease

**DOI:** 10.1093/lifemedi/lnae030

**Published:** 2024-08-31

**Authors:** Lin Sun, Zhensheng Yue, Lin Wang

**Affiliations:** Department of Hepatobiliary Surgery, Xi-Jing Hospital, Fourth Military Medical University, Changle West Road, Xincheng District, Xi’an, Shaanxi 710032, China; Department of Hepatobiliary Surgery, Xi-Jing Hospital, Fourth Military Medical University, Changle West Road, Xincheng District, Xi’an, Shaanxi 710032, China; Department of Ophthalmology, Xi-Jing Hospital, Fourth Military Medical University, Changle West Road, Xincheng District, Xi’an, Shaanxi 710032, China; Department of Hepatobiliary Surgery, Xi-Jing Hospital, Fourth Military Medical University, Changle West Road, Xincheng District, Xi’an, Shaanxi 710032, China

**Keywords:** epigenetics, nonalcoholic fatty liver disease, inflammation

## Abstract

Nonalcoholic fatty liver disease (NAFLD) is the most prevalent chronic liver condition, characterized by a spectrum that progresses from simple hepatic steatosis to nonalcoholic steatohepatitis, which may eventually lead to cirrhosis and hepatocellular carcinoma. The precise pathogenic mechanisms underlying NAFLD and its related metabolic disturbances remain elusive. Epigenetic modifications, which entail stable transcriptional changes without altering the DNA sequence, are increasingly recognized as pivotal. The principal forms of epigenetic modifications include DNA methylation, histone modifications, chromatin remodeling, and noncoding RNAs. These alterations participate in the regulation of hepatic lipid metabolism, insulin resistance, mitochondrial injury, oxidative stress response, and release of inflammatory cytokines, all of which are associated with the onset and progression of NAFLD. This review discussed recent advances in understanding the potential epigenetic regulation of inflammation in NAFLD. Unraveling these epigenetic mechanisms may facilitate the identification of early diagnostic biomarkers and the development of targeted therapeutic strategies for NAFLD.

## Introduction

Approximately 25% of adults worldwide suffer from nonalcoholic fatty liver disease (NAFLD), making it the most prevalent chronic liver condition. NAFLD can progress from simple steatosis to nonalcoholic steatohepatitis (NASH), which is characterized by inflammation and fibrosis, and may further advance to cirrhosis and hepatocellular carcinoma (HCC), among other severe conditions [1, 2]. The accumulation of hepatic fat initiates a cascade of signaling events that disrupt lipid and glucose metabolism. This disruption is evidenced by the accumulation of intracellular fat vesicles, impaired mitochondrial β-oxidation, increased oxidative stress, activation of pro-inflammatory pathways, and eventual hepatocyte apoptosis [3, 4]. Inflammation is a critical driver in the progression of liver fibrosis in NAFLD. However, assessing inflammation in NAFLD is challenging due to the disease’s dynamic nature and the limited correlation with conventional liver biochemical markers.

Epigenetics explores how external factors like the environment and lifestyle can impact gene expression and cellular characteristics without altering the DNA sequence. This field involves various molecular modifications on the genome, such as DNA methylation, histone modifications, chromatin remodeling, and noncoding RNAs, which can influence gene expression patterns independently of changes to the DNA sequence. These epigenetic alterations are critical in processes like cell differentiation, tissue development, and disease formation. Studies suggest that the advancement of the disease is aided by systemic metabolic inflammation, particularly that which results from a disruption of the metabolism of adipose tissue. Metabolic disorders associated with NAFLD are caused by environmental variables and epigenetic alterations [5, 6]. Thus, epigenetic changes may also contribute to the development and progression of NAFLD. For instance, abnormal levels of DNA methylation and histone modifications are linked to NAFLD onset and progression. So as to individual differences in genetic, nutritional, behavioral, and environmental factors that contribute to NAFLD, genetics is an intrinsic aspect that cannot be disregarded [7, 8]. Consequently, understanding epigenetics can shed light on the underlying mechanisms of NAFLD and aid in the development of therapeutic interventions.

NAFLD has garnered significant attention in recent research due to its critical role in inflammation, tissue regeneration, cancer development, and epigenetic regulation researches. This article will focus on the epigenetic mechanisms that are crucial to the pathophysiology of NAFLD. Given the pivotal role of inflammation in the development of hepatocarcinogenesis, we will also emphasize the role of epigenetic regulation in NAFLD-mediated inflammation.

## NAFLD and inflammation

The prevalence of NAFLD, a significant global health burden, is rising [9]. Although NAFLD is fundamentally a metabolic illness, it also involves various inflammatory processes mediated by immune cells, particularly when it progresses to NASH. At this stage, inflammation plays a critical role in the advancement of the disease [10, 11]. The current status and ongoing evolution of hepatic immune cells during NASH directly affect disease severity. Limiting the onset of NASH and preventing its progression to liver failure and HCC is a major global health challenge. With the ongoing identification of inflammatory markers, early detection of patients with NAFLD is a key challenge in managing this disease. This early detection is also crucial for improving patient selection and the design of clinical trials for emerging drug therapies. The understanding of the lipotoxicity process is one of the major developments that aids in the explanation of NAFLD in patients [12]. In fact, the kind of fat that builds up during hepatic lipid overload and the way hepatocytes handle this lipid load can either cause isolated hepatic steatosis to adapt or they can cause cell death through a variety of different molecular mechanisms. The latter causes the hepatocytes to release stress signals, also known as danger signals, which in turn activate aseptic inflammatory pathways [13, 14]. If these processes continue over time, they can result in an abnormal wound-healing response that causes chronic injury and fibrosis.

A popular area of study in recent years has been the function of the innate immune response in NAFLD. Changes in microbial homeostasis and/or bacterial translocation are linked to the activation and recruitment of hepatic immune cells via local signals, signals from adipose tissue, or signals from the gut. These events may trigger an inflammatory response that results in cellular damage and death, hence accelerating the progression of NAFLD disease [15–18]. The present research demonstrates the significance of intracellular or surface-expressed pattern recognition receptors in identifying pathogen invasion and cell injury in NAFLD/NASH [19]. One of the key mechanisms of liver injury in liver disease (particularly NASH) is aseptic inflammation, which happens in the absence of pathogens or external antigens and can result in chronic inflammation and the advancement of the illness. A class of intracellular molecules known as damage-associated molecular patterns (DAMPs) are produced or secreted when a cell is injured or dies, and they seem to be important triggers of sterile inflammation [20]. Numerous molecules, such as nuclear factors like high mobility group box 1, nuclear and mitochondrial DNA, purine nucleotides (adenosine triphosphate (ATP) and uridine triphosphate (UTP)), and uric acid, have been identified as DAMPs, However, it is unclear how much of each molecule contributes to inflammation in liver disease [21, 22]. Pathogen-associated molecular patterns (PAMPs) are implicated in liver injury in NAFLD/NASH in addition to DAMPs. When the intestinal mucosal barrier is compromised, bacterial products such as bacterial lipopolysaccharides from Gram-negative bacteria’s cell walls, as well as other molecules like peptidoglycan, bacterial lipoproteins flagellin, bacterial RNA, and DNA, etc., can reach the liver and activate innate immune cells locally, resulting in an intracellular signaling cascade response that amplifies the damage [23–25]. DAMPs and PAMPs cause a localized inflammatory response, which sets off a cycle of damage amplification that eventually damages organs. This is primarily caused by the generation of pro-inflammatory cytokines including interleukin-6 (IL-6) and tumor necrosis factor-α (TNF-α), which have significant metabolic effects on insulin resistance and lipid metabolism in addition to inducing inflammation [26, 27]. The majority of hepatocytes, including hepatocytes, Kupffer cells (KCs), hepatic stellate cells (HSCs), biliary epithelial cells, and sinusoidal endothelial cells, express the toll-like receptors, which are the best-characterized type of peripheral blood receptors and are expressed in different patterns in each of these cell populations [28–30].

We have tried to discuss the role of epigenetic modification-mediated inflammation in NAFLD here, especially in light of the recent advancements in the field. Although it is acknowledged that chronic inflammation plays a role in the onset and progression of NAFLD, the precise underlying mechanisms remain unclear, and there is not enough literature to discuss this significant and fascinating new research area.

## Epigenetics of NAFLD

**Figure 1.**
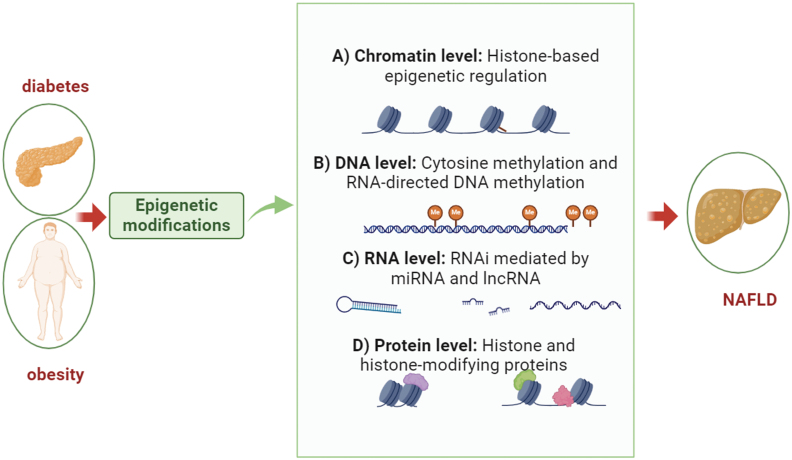
**Brief information on the epigenetics of NAFLD.** The figure highlights four major metabolic diseases influenced by epigenetic regulation, including diabetes and its complications, obesity, and NAFLD.

Epigenetics plays a crucial role in the complex interplay between environmental factors and genetics [31], which is common in NAFLD, the hepatic manifestation of the metabolic syndrome. Inflammatory processes in NAFLD can be affected by epigenetic phenomena such as DNA methylation, histone modifications, chromatin remodeling, and noncoding RNA expression [32]. A summary of the epigenetics of NAFLD can be found in Fig. 1.

### Methylation of DNA and NAFLD inflammation

DNA methylation is a modification that occurs on cytosine nucleotides, particularly those preceding guanine, known as CpG islands. Hypermethylation of CpG islands is associated with gene inactivation or silencing, whereas hypomethylation of CpGs promotes gene activation [33–35]. Hepatic steatosis is a key component of NAFLD pathophysiology [36], requiring the coordination between peroxisome proliferator-activated receptor alpha (PPARα) and PPARγ to balance fatty acid synthesis and oxidation. PPARα regulates peroxisomal and mitochondrial β-oxidation in the liver and skeletal muscle, while PPARγ plays a crucial role in adipogenesis and reducing the expression of pro-inflammatory cytokines. In NAFLD, PPARγ activation is linked to promoting lipid storage in adipose tissue rather than in the liver, potentially offering protection against hepatic steatosis. Modulating PPAR activity through pharmacological agents or lifestyle interventions has been explored as a potential therapeutic strategy for NAFLD. Lipogenesis is promoted by the downregulation of PPARα expression in hepatic steatosis, as reported [37]. Furthermore, methylation levels of the PPARγ coactivator 1α promoter correlated with homeostasis models assessing insulin resistance and fasting insulin, whereas methylation levels of the mitochondrial transcription factor A promoter negatively correlated with fasting insulin [38, 39]. These epigenetic changes in hepatic PPARγ in NAFLD patients have also been demonstrated to contribute to insulin resistance.

Furthermore, significant methylation of mitochondrial NADH dehydrogenase 6 (MT-ND6) has been demonstrated to result in the down-regulation of mitochondrial MT-ND6 mRNA in patients with NASH. DNA methylation has also been implicated in the pathogenesis of NAFLD and its progression to the fibrotic stage [40]. In human liver samples, collaborative global studies of gene expression and methylation have revealed that advanced disease at several CpG loci is consistent with regional dysregulation of methylation (mostly hypomethylation). These studies have also shown that tissue architecture, repair, and oncogenesis are associated with hypomethylation and enhanced gene expression, while essential metabolic functions, such as mitochondrial lipid metabolism, urea cycle, and amino acid biogenesis, as well as genes belonging to the P450 cytochrome family, are associated with hypermethylated transcriptional repressor genes [37, 41].

Growing evidence has connected hepatic DNA methylation and insulin tolerance to the conversion of simple steatosis into fibrosis in NASH [42]. This systematic histology analysis is gradually revealing the essential role of DNA methylation in the development of NAFLD. A new methylation and transcriptome analysis suggests that differentially methylated genes will explain the differences between patients with advanced NASH and simple steatosis [43].

### Histone modifications and NAFLD inflammation

Histone modifications play a crucial role in NAFLD. Research indicates a close association between abnormal changes in histone modifications and the onset and progression of NAFLD. For instance, some studies have observed abnormal levels of histone acetylation and methylation in liver tissues of NAFLD patients. These aberrant histone modifications may alter gene expression patterns, thereby influencing the pathogenic mechanisms of NAFLD such as fat accumulation, inflammatory responses, and cellular apoptosis. Therefore, investigating the role of histone modifications in the pathogenesis of NAFLD contributes to a deeper understanding of its development and may offer novel targets and strategies for NAFLD treatment.

Histones undergo multiple modifications, such as acetylation, phosphorylation, methylation, ubiquitination, SUMOylation, and ribosylation, with acetylation being the most extensively studied [44]. Changes in histone modifications may lead to dysregulation of various biological processes associated with NAFLD, including hepatic lipid accumulation, endoplasmic reticulum (ER) stress, oxidative stress, mitochondrial impairment, and inflammation. These processes can operate individually as distinct mechanisms or synergistically with environmental factors, collectively influencing the development of NAFLD [45]. Histone acetylation, which is caused by histone acetyltransferases (HAT), encourages gene transcription, while histone deacetylation, which is caused by histone deacetylases (HDAC), results in gene silencing. Transcriptional coactivators are essential components of transcriptional regulators that regulate inflammatory processes focused on nuclear factory in the HAT family member p300. Poor blood glucose regulation enhances the function of nuclear factor-κB (NF-κB) and generates pro-inflammatory genes between HAT and nuclear factor-β, such as p300 [46]. The majority of HDACs, including HDAC3, whose genomic recruitment influences glucose metabolism and circadian rhythms, are believed to be crucial in the development of NAFLD. Defects in the regulation of HDAC3 can result in irregular lipid metabolism in the liver, which in turn promotes NAFLD [47].

### Chromatin remodeling and NAFLD inflammation

There is a notable correlation between chromatin remodeling and inflammation in NAFLD. Research indicates that the single-stranded DNA-binding protein replication protein A1 (RPA1) can bind to gene regulatory regions, chromatin remodeling factors, and HNF4A, thereby reshaping chromatin structure. Moreover, RPA1 enhances the transcriptional activity of HNF4A and promotes fatty acid β-oxidation through this chromatin structure [48]. Chromatin remodeling can affect the regulation of gene expression, including genes related to inflammation. During the development of inflammation in NAFLD, chromatin remodeling may be involved in the following ways: chromatin remodeling can influence the expression of inflammation-related genes, such as pro-inflammatory and anti-inflammatory factors. This may lead to enhanced or weakened inflammatory responses, thereby affecting the severity of inflammation in NAFLD [49]. Chromatin remodeling can also impact the function and activity of immune cells, thus influencing the extent and duration of inflammatory responses. Chromatin remodeling may also modulate the activity of cell signaling pathways, such as the NF-κB signaling pathway, thereby regulating the occurrence and development of inflammation [50].

Therefore, the correlation between chromatin remodeling and inflammation in NAFLD primarily manifests in the regulation of inflammatory gene expression and the modulation of inflammatory-related cytokines and signaling pathways. In-depth research into the role of chromatin remodeling in the development of inflammation in NAFLD can contribute to a better understanding of the pathogenesis of NAFLD and provide new insights for the development of related therapeutic strategies.

### Noncoding RNAs and NAFLD inflammation

#### miRNAs in NAFLD inflammation

Among the several noncoding RNA subtypes, microRNA (miRNA) has been extensively researched. Several reports have connected these tiny molecules to the control of inflammation in NAFLD [51]. miRNAs are a family of endogenous, noncoding functional RNAs that have a maximum length of about 22 nucleotides. They regulate gene expression by preventing or reducing the translation of target genes, but they also play a role in cell formation, proliferation, differentiation, and apoptosis [52].

**Figure 2.**
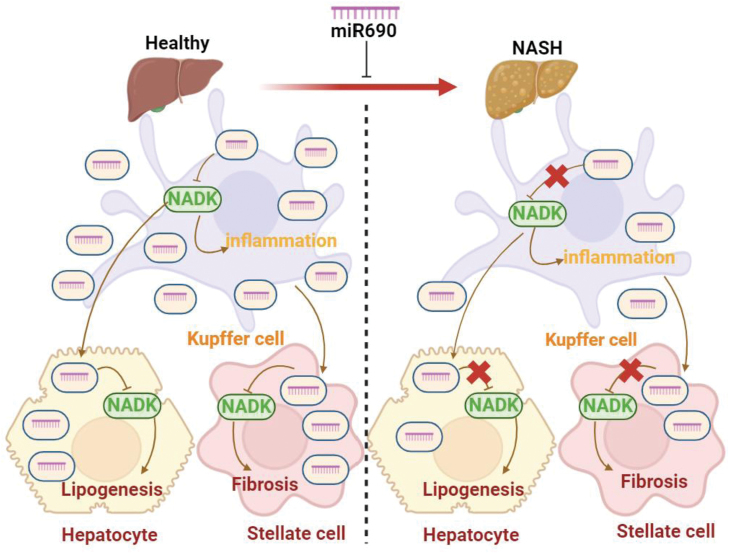
**The role of miR-690 of inflammation in NASH.**Endogenous miR-690 is produced by KCs and shuttles this miRNA to other hepatocytes such as hepatocytes, and miR-690’s primary target is NADK mRNA, and NADK levels are inversely correlated with cellular miR-690 content.

KCs generate miR-690, which is subsequently secreted by exosomes to other hepatocytes, including recruited hepatic macrophages and HSCs. In HSCs, miR-690 directly suppresses adipogenesis, inflammation, and fibrosis. When miR-690 mimics were given to NASH mice, all aspects of the NASH phenotype were significantly inhibited, leading to miR-690 deficit in KCs throughout the development of NASH. Because miR-690’s KC-specific KOs increase NASH development, miR-690 levels in mouse and human NASH livers were considerably lower than those of controls. NAD kinase (NADK) mRNA is the principal target of miR-690, the amount of NADK in cells is inversely associated with the amount of miR-690. In Fig. 2, the role of miR-690 in NASH is summarized. According to these researches, KCs are crucial to the etiology of NASH, which also suggests that the inhibition of the inflammatory program inside these immune cells is the cause of the decrease in inflammatory gene expression. and suggest that miR-690 might be a useful treatment for this illness [53].

**Figure 3.**
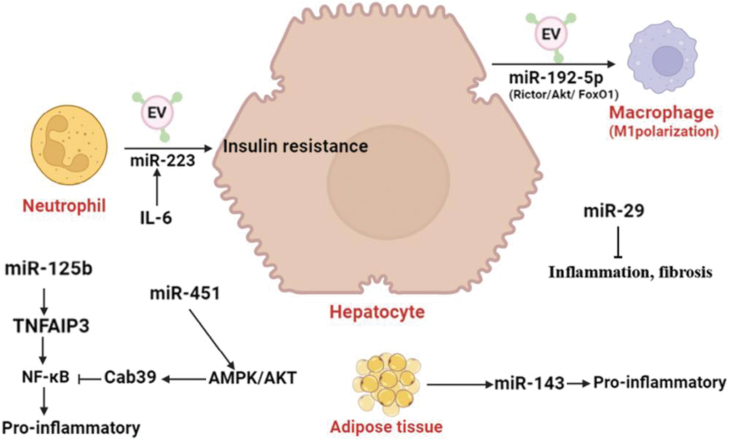
**The role of partial miRNA in NAFLD inflammation.**miR-192-5p plays a key role in pro-inflammatory macrophage activation and disease progression in NAFLD by regulating Rictor/Akt/FoxO1 signaling. miR-451 can activate the AMPK/AKT pathway, thereby reducing hepatic inflammation by targeting Cab39 to inhibit NF-κB activation.

Furthermore, a few miRNAs that control lipid metabolism are also implicated in inflammation. For instance, the Rictor/Akt/FoxO1 pathway can be used by exosomal miR-192-5p from NAFLD hepatocytes to stimulate macrophage activation [54]. Serum exosomal miR-192-5p is a potential noninvasive biomarker and therapeutic target for NAFLD/NASH. In addition, miR-125b can enhance NF-κB signaling by targeting tumor necrosis factor alpha-induced protein 3, thereby exacerbating the inflammatory response [55, 56]. In addition, miR-223 plays a crucial role in the crosstalk between inflammatory cells and hepatocytes via extracellular vesicles (EVs). It has been reported that IL-6 stimulates myeloid cells to release miR-223-containing exosomes, and miR-223 is taken up by hepatocytes, which can take up neutrophil-derived EVs enriched with miR-223 in an apolipoprotein E-dependent manner [57, 58]. It has been reported that miR-451 can activate the AMP-activated protein kinase (AMPK)/protein kinase B (AKT) pathway, thereby reducing hepatic inflammation by targeting Cab39 to inhibit NF-κB activation [59]. miR-29 has been found to reduce hepatic inflammation and fibrosis after liver injury. Meanwhile, DNA methyltransferases have been reported to be involved in the development of nonalcoholic steatohepatitis [60, 61]. Lastly, increased expression of miR-143 was also noted. miR-143 is implicated in the control of cell proliferation, angiogenesis, apoptosis, and inflammation in adipose and vascular tissues [62]. Brief information on the above miRNA has been summarized in Table 1. The role of partial miRNA in NAFLD inflammation has been summarized in Fig. 3.

**Table 1.** miRNAs in NAFLD inflammation.

microRNA	Dysregulation	Disease output	Experimental model	References
miR-690	Upregulation	NASH	Rats	[45]
miR-192-5p	Upregulation	NAFLD	Human	[46]
miR-125b	Upregulation	Hepatic inflammation	Human	[48]
miR-223–3p	Upregulation	Hepatic insulin resistance and fatty acid metabolism in NAFLD	Rats	[49, 50]
miR-451	Downregulation	NAFLD	Mouse	[51]
miR-29 (a,b,c)	Upregulation	NAFLD	Human, *in vitro* (HEPG2 cell line)	[52, 53]
miR-143	Upregulation	Increase the inflammation	Human	[54]

To summarize, this section delves into the regulation mechanisms of several miRNAs in NAFLD/NASH inflammation and provides a quick overview of their expression levels in the liver and peripheral circulation. Since NAFLD/NASH patients’ serum levels of certain miRNAs, like miR-21 and miR-192, differ considerably from those of healthy controls, these miRNAs may be employed as biomarkers for noninvasive diagnosis. Through a number of common pathways linked to inflammation, including the TLR4 and NF-κB/TNF-α pathways, miRNA plays a role in the development of inflammation. As a result, decreasing inflammation is a useful tactic to stop NAFLD from progressing, and the previously described miRNA may offer a theoretical framework for miRNA-based illness treatment.

#### Long noncoding RNAs in NAFLD inflammation

Long noncoding RNAs (lncRNAs) are noncoding RNAs consisting of more than 200 nucleotides with little or no protein-coding ability [63]. Novel lncRNA nuclear episomal assembly transcript 1 (NEAT1) is transcribed from several loci associated with endocrine tumors [64]. In sepsis-induced liver injury, NEAT1 has been shown to enhance inflammatory responses. Moreover, NEAT1 has been shown to increase hepatic lipid buildup and worsen NAFLD [65, 66]. Liu et al. noted increased expression of lncRNA zinc finger antisense 1 (ZFAS1) in NAFLD patients, and the high expression level may be considered as a risk factor for NAFLD, and elimination of ZFAS1 improves inflammation and oxidative stress indices [67]. Ye et al. discovered that 381 lncRNAs are more abundant in NASH as well as significantly expressed in NAFLD. Both the NASH cell model and the NASH animal model’s liver tissues showed noticeably high expression of a new lncRNA called gm9795. It was discovered that gm9795 markedly increased the expression of TNF-α, IL-6, and IL-1β, three crucial inflammatory mediators in NASH, in the NASH cell model. They also discovered that the NF-κB/JNK pathway was activated and that gm9795 upregulated important molecules in ER stress [68]. Steroid receptor RNA activator (SRA) is an interesting lncRNA that is closely linked to inflammation during development of NAFLD. It has been demonstrated to have an impact on diet-induced obesity, glucose tolerance, and hepatic steatosis. *In vitro* and *in vivo* expression of inflammatory genes is decreased by SRA deficiency. Aerobic exercise has been demonstrated to dramatically inhibit SRA and lower the expression of genes associated with pro-inflammatory factors; the P38/c-Jun N-terminal kinase (JNK) signaling pathway has also been reported to exhibit similar significant changes. The process was clarified by demonstrating that SRA suppresses the transcriptional activity of forkhead box protein O1 (FoxO1) in hepatocytes through an insulin-independent pathway. This lowers the expression of its downstream gene, adipose triglyceride lipase, an important lipolytic enzyme, and consequently lowers the β-oxidation of free fatty acids in the liver. According to the above, the JNK/P38 pathway, the ER stress of IRE1α, and PKR-like endoplasmic reticulum kinase (PERK) transactivation are closely associated to the inhibition of SRA expression by aerobic exercise in hepatic steatosis to reduce inflammation [69].

Moreover, it has been demonstrated that metastasis-associated lung adenocarcinoma transcript 1 (MALAT1) propagates inflammation and fibrosis through its interaction with SIRT1. There is an overexpression of lnc-MALAT1 in liver damage. When lnc-MALAT1 attaches to SIRT1, it inactivates SIRT1, which then activates HSC, causing extracellular matrix deposition and fibrosis [70]. In HepG2 cells, a new intergenic lncRNA identified RP11-91K9.1 and situated on chromosome 3q26.32 exhibited the highest sensitivity to TNF-α stimulation. The researchers called this lncRNA lncTNF because it showed a favorable correlation with lobular inflammation in human liver tissues. Remarkably, they discovered that lncTNF responded to pro-inflammatory stimuli like interleukin 1β (IL-1β). TNF-α and IL-1β both trigger the NF-κB pathway, indicating that lncTNF plays a part in inflammatory responses in the liver via NF-κB signaling [71].

**Figure 4.**
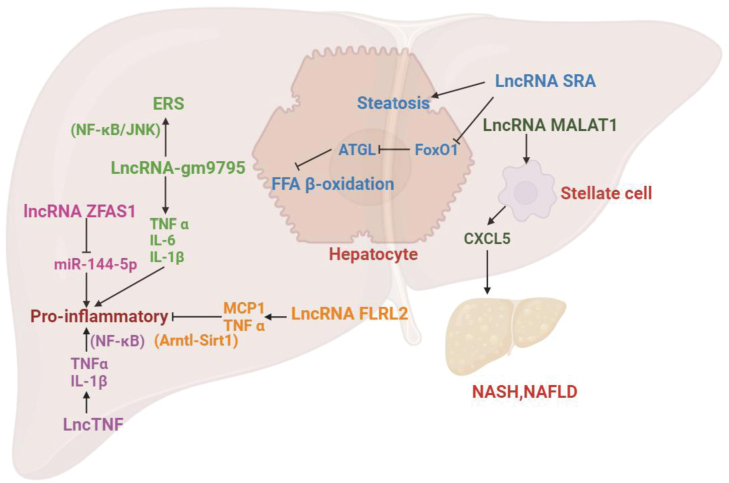
**The role of these lncRNAs in NAFLD inflammation. **lncRNA-Gm9795 promoted the expression of TNF-α, IL-6, and IL-1β, important inflammatory mediators in NASH. lnc-MALAT1 binds to SIRT1, leading to its inactivation and subsequent activation of HSC and resulting in extracellular matrix deposition and fibrosis. lncRNA-FLRL2 activated the Arntl-Sirt1 axis, and suppressed adipogenesis, ER stress, and inflammation.

Lastly, FLRL2, or fatty liver-associated lncRNA 2, may serve as a crucial regulator of NAFLD. To explore the potential therapeutic benefits of FLRL2 intervention in NAFLD, researchers developed an overexpression model of FLRL2 in NAFLD mice. The results showed a significant reduction in hepatic steatosis, activation of the Arntl-Sirt1 axis, and suppression of adipogenesis, ER stress, and inflammation. These findings provide initial evidence supporting the therapeutic potential of FLRL2-mediated gene modification in NAFLD and underscore the prospective utility of targeting FLRL2 and the Arntl-Sirt1 axis for NAFLD treatment [72]. Table 2 summarizes key information regarding the discussed lncRNAs. The role of these lncRNAs in NAFLD-related inflammation is illustrated in Fig. 4.

**Table 2.** lncRNAs in NAFLD inflammation.

lncRNAs	Target genes	Signaling pathways	References
NEAT 1	miR-146a-5p	Let-7a/TLR4	[57]
lncRNA ZFAS1	miR-144-5p	Unknown	[59]
lncRNA-Gm9795	TNF-α，IL-6，and IL-1β	NF-κB/JNK	[60]
lncRNA SRA	FoxO1	FFA β-oxidation	[61]
lncRNA MALAT1	CXCL5	Extracellular space	[62]
lncTNF	TNF-α and IL-1β	NF-κB	[63]
lncRNA FLRL2	MCP1 and TNF-α	Arntl-Sirt1	[64]

#### Circular RNAs in NAFLD inflammation

Circular RNAs (circRNAs) were first identified using electron microscopy in 1976 in plant-like viruses. In 1979, they were found in the cytoplasm of HeLa cells, a type of eukaryotic cell line. The unique covalently closed loop structure of circRNAs enhances their stability and protects them from degradation by nucleic acid exonucleases due to the absence of polyadenylation at their 3ʹ and 5ʹ ends [73]. CircRNAs are known to play a significant role in cancer biology [74], but their function in NASH remains largely unexplored. Overexpression of circRNA_0001805 has been shown to inhibit liver inflammation induced by a HFD [75]. In another recent study, circRNA_1639 was found to activate inflammatory responses by acting as a sponge for miR-122 in the liver and hepatic macrophages. However, further research is needed to confirm if circRNA_1639 plays a similar role in the inflammatory pathways of NASH, as these findings were derived from studies on mice livers in the context of alcoholic liver injury, which may not fully apply to NASH-related injury [76]. Table 3 provides a summary of the key information regarding the circRNAs discussed above.

**Table 3.** CircRNAs in NAFLD inflammation.

CircRNAs	Target genes	Signaling pathways	References
circRNA_0001805	TNF-α, IL-6, and IL-1β	NF-κB	[67]
Circ_PSD3	miR-122	Unknown	[68]

## Epigenetics and potential clinical perspective

Research into the modulation of NAFLD development of inflammation by epigenetic changes, is still in its early stages and faces numerous challenges. Evidently, various factors are involved in different stages of NAFLD. However, the interactions among these factors and their impact on metabolic homeostasis remain unclear [77]. As of now, the US FDA has not approved any epigenetic drugs specifically for treating NAFLD and other metabolic diseases. Furthermore, effective diagnostic and therapeutic targets for NAFLD are scarce. The complex molecular interactions and overlapping pathways in NAFLD patients may pose difficulties [78]. However, several potential epigenetic drugs are already being tested in clinical trials to treat NAFLD, such as curcumin (histone acetyltransferase inhibitor), resveratrol (sirtuin-activating compound), ISIS 703802 (antisense oligonucleotide).

In NASH-related tumor patients, changes in DNA methylation of certain tumor-related genes and alterations in gene expression are associated with necroinflammatory grading, a characteristic of NASH, and are linked to poorer tumor differentiation [40]. This suggests that NASH-specific DNA methylation features play a role in the development of NASH-related hepatocellular carcinoma by altering the expression of tumor-related genes. Extensive research indicates that functionally relevant methylation differences can stratify NAFLD patients. Genes with DNA methylation changes are associated with the incidence and progression of NAFLD, regulating processes such as steatohepatitis, fibrosis, and carcinogenesis, highlighting the role of DNA methylation in NAFLD progression and its clinical significance for diagnosis, prognosis, and therapeutic intervention [79].

Moreover, the use of miRNAs as important biomarkers for NAFLD still has limitations. To effectively utilize miRNA panels as molecular biomarkers, standardized methods for evaluation and validation using larger cohorts are required [80]. Growing research indicates that lncRNAs are important modulators of gene expression via the regulation of several cellular functions and are connected to the etiology of multiple human illnesses, such as cancer, metabolic diseases, and neurodegenerative disorders [81]. As the field of lncRNAs develops, it is expected that they will elucidate many of the “missing pieces” in the molecular pathways that regulate metabolic control and, when dysfunctional, contribute to the development of metabolic diseases [82]. However, to date, most of the experimental focus on lncRNAs has centered on large-scale discovery based on genome-wide approaches, the functionality of lncRNAs remains understudied. In conclusion, while miRNAs and lncRNAs hold promise as biomarkers and regulators in NAFLD, further research is needed to overcome current limitations and fully understand their roles and potential clinical applications.

## Conclusions

In this review, we attempt to summarize the role of epigenetics in NAFLD inflammation and progression. Currently, this emerging class of epigenetics is involved in the critical regulation of various biological and pathophysiological processes. Being present in 70% of type 2 diabetics and 80% of obese individuals, NAFLD is the most common chronic liver disease and is intimately linked to the global obesity and type 2 diabetes mellitus (T2DM) epidemics [83]. Insulin resistance contributes to the development of prediabetes and its progression to T2DM, as well as to the spontaneous onset of NAFLD, and clinical studies on the effect of metformin use on the incidence and prognosis of HCC in patients with NAFLD/NASH have begun to emerge, but remain understudied [84]. NAFLD does not currently have a specific treatment. Although they can assist halt the advancement of the condition, current treatment modalities—such as weight loss and lifestyle modification—are ineffectual because of low patient compliance. Antidiabetic medications are commonly used to treat NASH, yet they do not explicitly address liver damage and are not curative in the majority of patients. The need for new NASH medicines to be developed grows as the prevalence rises. In this review, we highlight the critical role that epigenetics play in inflammation along the course of NAFLD development. Unlike genetics, epigenetic alterations are mostly reversible, hence, epigenetics has great potential clinical application. In addition, several epigenetic drugs have entered clinical trials to treat NAFLD.

Consequently, as we have covered, epigenetics plays a significant part in the pathophysiology of NASH. These epigenetic alterations hold potential as diagnostic, prognostic, and therapeutic monitoring markers, offering novel avenues for personalized medicine and precision treatment of NAFLD. With the advancement of bioinformatics, coupled with breakthroughs in technologies such as gene editing and high-throughput sequencing, the field of epigenetics has seen rapid progression. Despite numerous studies on epigenetics in NAFLD, significant challenges remain. Research into the role of chromatin remodeling in NAFLD is still in its nascent stages. While numerous studies have explored the potential of DNA methylations, noncoding RNAs and histone modifications, transitioning these findings into clinical settings for diagnosis and treatment presents substantial challenges. This disconnect underscores the complexity of translating molecular mechanisms into practical, therapeutic strategies and points to the need for further targeted research to bridge this gap effectively. To date, no epigenetic drug has been approved for the treatment of NAFLD. Moreover, there are concerns about the potential adverse effects of epigenetic drugs, particularly their capacity to alter off-target genes. However, by delving into the specific mechanisms and clinical implications of epigenetics in NAFLD, it is anticipated to achieve new breakthroughs and advancements in its diagnosis, treatment, and prevention. Nonetheless, comprehensive clinical studies and validations are required to fully uncover the potential of these epigenetic modifications in NAFLD.
